# A Comparative Analysis of the Cytotoxic and Vascular Activity Effects of Western Diamondback Rattlesnake (*Crotalus atrox*) and Eastern Diamondback Rattlesnake (*Crotalus adamanteus*) Venoms Using a Chick Embryo Model

**DOI:** 10.3390/ani14111634

**Published:** 2024-05-30

**Authors:** Barbora Bekešová, Vladimír Petrilla, Magdaléna Polláková, Zuzana Andrejčáková, Radoslava Vlčková, Barbara Dyba, Drahomíra Sopková, Monika Petrillová, Eva Petrovová, Jaroslav Legáth

**Affiliations:** 1 Department of Biology and Physiology, University of Veterinary Medicine and Pharmacy, Komenského 73, 041 81 Košice, Slovakia; vladimir.petrilla@uvlf.sk (V.P.); magdalena.pollakova@uvlf.sk (M.P.); zuzana.andrejcakova@uvlf.sk (Z.A.); radoslava.vlckova@uvlf.sk (R.V.); drahomira.sopkova@uvlf.sk (D.S.); 2 Zoological Department, Zoological Garden Košice, Široká 31, 040 06 Košice-Kavečany, Slovakia; 3 Department of Biochemistry and Biophysics, University of the National Education Commission, Podchorążych 2 Street, 30-084 Cracow, Poland; barbara.dyba@uken.krakow.pl; 4 Department of General Competencies, University of Veterinary Medicine and Pharmacy, Komenského 73, 041 81 Košice, Slovakia; monika.petrillova@uvlf.sk; 5 Department of Morphological Disciplines, University of Veterinary Medicine and Pharmacy, Komenského 73, 041 81 Košice, Slovakia; eva.petrovova@uvlf.sk; 6 Department of Pharmacology and Toxicology, University of Veterinary Medicine and Pharmacy, Komenského 73, 041 81 Kosice, Slovakia; jaroslav.legath@uvlf.sk; 7 Department of Biotechnology and Bioinformatics, Faculty of Chemistry, Rzeszow University of Technology, Powstańców Warszawy 6, 35-959 Rzeszów, Poland

**Keywords:** chorioallantoic membrane, snake venom, chicken embryo, CHEST, Crotalus, HET-CAM

## Abstract

**Simple Summary:**

Crotalus snake envenomation poses a serious challenge due to its diverse toxicological effects, including neurological, myotoxic, and cytotoxic symptoms, often leading to death. The aim of the study was to elucidate the physiological effects of exposure to *Crotalus atrox* and *Crotalus adamanteus* venoms and to assess toxicity using chicken embryo models. Currently, there is not a lot of research demonstrating the physiological effects of venom, including its potential impact on embryos, in accordance with the 3R rules. The applied research model consisted of the chick embryotoxicity screening test (CHEST) and the chick chorioallantoic membrane (CAM) test, which allowed for (i) a demonstration of the greater toxicity of *C. adamanteus* venom and (ii) an observation of the embryotoxic effect and vasoactive nature of the tested venom species. Additionally, (iii) morphological abnormalities (such as Siamese twins) emerged, and (iv) changes in the activity of acetylcholinesterase (AChE) were identified (resulting solely from its presence in the examined tissues due to the lack of this component in exogenously applied venom). These results provide crucial insights into the mechanisms of Crotalus venom toxicity and their potential biomedical applications.

**Abstract:**

Crotalus snakebites induce various toxicological effects, encompassing neurological, myotoxic, and cytotoxic symptoms, with potentially fatal outcomes. Investigating venom toxicity is essential for public health, and developing new tools allows for these effects to be studied more comprehensively. The research goals include the elucidation of the physiological consequences of venom exposure and the assessment of toxicity using animal models. Chicken embryos serve as valuable models for assessing venom toxicity through the chick embryotoxicity screening test (CHEST) and the chick chorioallantoic membrane (CAM) assay, particularly useful for evaluating vascular impacts. *C. adamanteus* venom application resulted in higher embryotoxicity and morphological abnormalities, such as Siamese twins. The CAM assay demonstrated the hemorrhagic effects of venom, varying with venom type and concentration. The irritant potential of both venom types was classified as slight or moderate depending on their concentration. Additionally, acetylcholinesterase (AChE) activity was performed to receive information about organ toxicity. The results show that both venoms induced changes in the whole embryo, heart, and liver weights, but the *C. adamanteus* venom was identified as more toxic. Specific venom concentrations affected AChE activity in embryonic tissues. These findings underscore the embryotoxic and vasoactive properties of *Crotalus venoms*, providing valuable insights into their mechanisms of toxicity and potential applications in biomedicine.

## 1. Introduction

The Crotalus genus comprises a group of venomous pit vipers, commonly known as rattlesnakes or rattlers, belonging to the viper family and naturally found in the Americas [1,2,3,4]. The western diamondback rattlesnake (*Crotalus atrox*) and the eastern diamondback rattlesnake (*Crotalus adamanteus*) represent species of this genus occupying distinct geographical niches [5,6]. Based on the taxonomic relationship between *Crotalus atrox* and *Crotalus adamanteus*, both species, like all rattlesnakes in this genus, possess potent venom designed to incapacitate prey [7]. Recent research suggests that despite the presence of many similar venom components in snakes of the Crotalus genus, different species within this group exhibit distinct venom compositions [8,9], further highlighting their intriguing characteristics. However, differences in morphology [10] and diet [11,12] reflect adaptations to specific environments and ecological niches, potentially influencing the modification of venom components.

Crotalus snake bites induce various toxicological effects, encompassing a spectrum of neurological, myotoxic, and cytotoxic symptoms, each characterized by unique physiological disruptions in tissues. At the clinical level, coagulopathy, hypertension, and renal and respiratory disturbances may occur, with potential anaphylactic or fatal consequences [13,14]. The western diamondback rattlesnake (*Crotalus atrox*), prevalent in North America, ranks among the most dangerous rattlesnake species in the region, believed to be responsible for a significant proportion of fatal snakebites in northern Mexico. In the United States, it trails only the eastern diamondback rattlesnake (*Crotalus adamanteus*) [5]. Similar to many other American viper representatives, the venom of Crotalus snakes contains numerous proteolytic enzymes, facilitating prey immobilization by breaking down structural tissues and proteins through catabolic processes. An enzyme in the diamondback rattlesnake venom, known as protease H, induces widespread bleeding. Five distinct proteolytic toxins from the western diamondback rattlesnake venom cause bleeding by disrupting laminin in band A within the basement membrane [15,16,17,18]. Additionally, snake venoms, including those from the Crotalus genus, primarily consist of proteins (approximately 95% of venom dry weight) with potential pharmacological properties, making them potential targets for various pharmacological studies [19,20]. The venom components of the Crotalus genus, like other zoonotic venoms, represent a source of potential biotechnological benefits due to their specialized and targeted interactions with numerous physiological targets [21]. While the World Health Organization (WHO) has classified snakebites as a neglected tropical disease [4,22], understanding the effects of these snake venoms on tissues is crucial not only from a clinical standpoint.

Elucidating the physiological consequences of venom exposure and assessing toxicity extent through animal model experiments are primary research goals. In this study, chicken embryos were utilized as a model organism, and the chick embryotoxicity screening test (CHEST), focusing on assessing substance effects on chick embryonic development, was employed to evaluate venom toxicity from representatives of the Crotalus genus. Chicken embryos are easily handled and maintained, with the ability for in ovo visual analysis post-venom applications, facilitating developmental monitoring throughout embryonic development. Data obtained in this manner can provide valuable insights into understanding the myriad anomalies observed in humans. Chicken embryos share morphological and biochemical similarities with mammalian embryos, rendering them ideal for determining teratogenic effects and studying venom effects not only during envenomation but also as a preclinical model for potential drug studies [23,24,25].

The chick chorioallantoic membrane (CAM) test, a widely used experimental model, investigates venom’s toxic effects directly on animal embryos, circumventing maternal variables (following the 3Rs rules). The CAM, a highly vascularized extraembryonic membrane, undergoes observable changes. The test involves applying substances, such as venoms, directly to the CAM surface, enabling the monitoring of their effects on angiogenesis and vessel integrity [26,27,28]. The CAM assay proves particularly useful in toxicology studies for assessing the vascular impacts of venom components or potential drug candidates. It is noteworthy that venom from *C. atrox* species primarily exhibits hemotoxic effects, targeting vascular structures, blood components, and cardiac tissue. Notably, venom components such as metalloproteinases play a pivotal role in hemorrhagic manifestations [14,29]. The study also measured the lethal dose of tested venoms on chicken embryos, defining potential toxic effects based on venom concentration correlation with subsequent mortality. Recent snake venom research indicates an active acetylcholinesterase (AChE) presence, although it is not reported in selected Crotalus species [9]. Hence, another significant goal of this study was to conduct an assay suitable for identifying potential neurotoxic venom effects—specifically, measuring AChE activity [28].

## 2. Materials and Methods

### 2.1. Venom Collection

Snake venom samples of two snake species, *Crotalus adamanteus* and *Crotalus atrox*, obtained from VIPERAFARM (Trnava, Slovakia; Cooperation Agreement No. 241/2017/UVLF) were used for the experiment. After venom extraction, which was induced with gentle pressure of a sterile jar with a membrane on the venom gland, the contents of the jar were dried thoroughly and carefully stored until further use. To avoid changes in the qualitative or quantitative composition of the venoms and consequent errors, the venoms were stored in a dark hermetically sealed container at a controlled temperature (4 °C).

### 2.2. The Chick Embryotoxicity Screening Test (CHEST)

A total of 117 chicken eggs (Lohmann Brown; the hatchery in Párovské háje, Nitra, Slovakia) were used for the CHEST. Eggs were divided equally into eight test groups and one control group (*n* = 13 for each group). The test groups were exposed to individual concentrations (2000, 200, 20, and 2 µg/mL) of both snake venoms. The venom concentrations were calculated based on the dissolution of lyophilized venom in a saline solution. The control group received equal amounts (100 µL) of sterile saline.

Fertilized hen eggs laid at approximately the same time were imported and stored at 15 °C until incubation. Before being placed in an automatic incubator (ET 49, ART 549/A), the eggs were cleaned with 70% ethanol and then incubated, blunt end up, at 37.5 °C and 60% humidity for 9 days. The incubation rack ensured that the eggs were rotated at regular 3 h intervals, preventing the embryos from adhering to the egg membranes.

The day the eggs were placed in the incubator was embryonic day zero, referred to as ED0 [26]. At ED4, eggs were removed from the incubator and the blunt end of each egg was washed with 70% ethanol. The disinfected area was then taped over with white adhesive tape and a hole was cut with scissors to allow for inspection of the inside of the egg, its vascular network, or the beating heart somewhere. Unfertilized eggs were discarded from the experiment at this stage.

Using the ‘window technique’, 100 µL of the appropriate venom concentrations (2000, 200, 20, and 2 µg/mL) were applied directly to the surface of the embryos on top of the inner shell membrane (*membrana papyracea*), while the control group was injected with the same amount of sterile saline (100 µL). After the application was complete, the holes on the eggs were hermetically sealed with transparent adhesive tape, labeled with the appropriate concentration of venom used, and placed back in the incubator where embryonic development continued.

Sampling was performed at ED9. The adhesive tape was removed from the eggs, and the individual holes were enlarged enough to allow for the embryos to be gently pulled out. Each test group, including the control group, was evaluated separately. The number of live and dead embryos in each group was monitored. The body weight of live embryos was recorded, as well as the possible occurrence of morphological alterations. Surgically dissected embryonic liver and heart tissues were weighed. The organs were then preserved at −80 °C until acetylcholinesterase activity analysis.

### 2.3. The Hen’s Egg Test on the Chorioallantoic Membrane (HET-CAM)

The HET-CAM assay is a method of monitoring and recording the onset of the first vasoactive effects (hyperemia, hemorrhage, and blood clots) of test substances on the surface of the chorioallantoic membrane (CAM) at precisely defined time intervals (0 s, 30 s, 120 s, 240 s) [30]. Based on Luepke’s system, 4 eggs were used for each concentration of the selected venom (see Figures S1–S4 in the Supplementary Materials).

On ED3, the eggs were removed from the incubator, and 70% ethanol was used to clean the spot at the sharp end of the egg, which was then perforated with sharp-tipped scissors. Through the created opening, 2 mL of white was removed from each egg using a syringe and a needle (20 G). After collection, the holes were sealed with hot paraffin, and all eggs were returned to the incubator where embryo development continued under unchanged conditions (temperature 37.5 °C, humidity 60%) [26].

Application of each venom concentration and evaluation of their effects was performed on ED9. The blunt ends of the eggs from the incubator were cleaned with 70% ethanol and taped over with adhesive tape to catch small pieces of shell falling into the interior of the egg later. Using sharp scissors, an opening was made in the blunt end of the egg large enough to allow safe removal of the shell from the shell membrane, exposing the CAM. At this stage, it was clear to identify eggs with live or dead embryos and select them from unfertilized eggs, the latter two mentioned being immediately discarded from the experiment.

The control group of eggs consisted of 4 randomly selected eggs that were administered 50 μL of sterile saline solution on the CAM surface. Similarly, for the individual test groups, 4 randomly selected eggs were used each time. In this experiment, two concentrations (2000 and 200 µg/mL) of each venom were used in this method; thus, a total of 20 eggs were used, including the control. Each egg was assessed individually in 5 min after the application of 50 μL of the substance to the surface of the CAM vessels. Photographs were taken at 0 s, 30 s, 120 s, and 240 s using a stereomicroscope (Olympus SZ61; Tokyo, Japan), a digital camera (ARTCAM-300MI, Artray Co., Ltd., Tokyo, Japan), and software (Quick Photo 2.3) to analyze the effects of the administered substances on the CAM surface [26].

### 2.4. Analysis of Acetylcholinesterase Activity

As the collection of embryo brains was not possible due to its size and hard separation from the surrounding tissues, acetylcholinesterase (AChE) activity analysis was performed on embryonic liver and heart tissue samples obtained from the preceding CHEST assay.

Samples preserved at −80 °C were thawed slowly under controlled conditions (approximately 25 °C) and then homogenized with a Sonoplus mini20 homogenizer (Bandelin, Berlin, Germany) in the buffer of the following composition: 100 mM Tris, 150 mM NaCl, 1 mM EGTA, 1 mM EDTA, 1% Triton X-100, 0.5% sodium deoxycholate, phosphatase inhibitor cocktail, protease inhibitor cocktail, and PMSF (pH 7.4; Sigma-Aldrich, St. Louis, MO, USA). Tissue homogenates (5 mg tissue/300 μL buffer) prepared were further analyzed with colorimetric supernatant acetylcholinesterase assay (Abcam, Shanghai, China) using an ELISA device (λ 410 nm; Multiskan^®^EX Spectrometer, Thermo-Fisher, Abingdon, UK). The activity of AChE, which hydrolyzes acetylthiocholine to thiocholine and acetate, was read on a microtiter plate, with subsequent quantification of thiocholine using the reagent DTNB (5,5′-dithiobis (2-nitrobenzoic acid; Abcam, Cambridge, UK), providing relative AChE activity, standardized to protein values (mU/mg protein) [28].

### 2.5. Statistical Analysis

The embryotoxic effects of selected snake venoms were statistically evaluated using an unpaired Student’s *t*-test (GraphPad Prism 5.0, San Diego, CA, USA). The values are given as mean ± SEM. Statistically significant changes from control are marked with an asterisk (* = *p* < 0.05; ** = *p* < 0.01; *** = *p* < 0.001).

## 3. Results

### 3.1. CHEST Test

An evaluation of the embryotoxic effects of snake venoms of the species *Crotalus adamanteus* and *Crotalus atrox* was performed on the 5th day after application (Table 1). In the control group where sterile saline was applied to the surface of embryos, there were no dead embryos found. The toxicity rate was almost half as high in embryos where the venom of *C. adamanteus* was applied compared to embryos with the applied venom of *C. atrox*. Out of the total 104 embryos to which venom was administered, 13 embryos were dead after venom application, of which 9 were dead after the application of *C. adamanteus* venom and 4 after *C. atrox* venom administration.

Based on the relationship between the venom concentrations used and subsequent mortality, LD_50_ values were determined for the two selected venoms. A higher mortality rate was observed after the application of *C. adamanteus* venom with LD_50_ = 3.96 μg/egg compared to LD_50_ = 11.73 μg/egg after the application of *C. atrox* venom.

The total embryo weights on embryonic day (ED) 9 as well as the weights of the organs (hearts and livers) obtained were compared with the control group (Table 2) as follows:

The whole embryo weights were observed as lower at the highest concentration (2000 µg/mL; *p* < 0.05) of *C. adamanteus* venom in comparison to the control. On the other hand, higher embryo weights compared to the control were recorded at all concentrations of the *C. atrox* venom (*p* < 0.01 for 2000 µg/mL and *p* < 0.001 for 200, 20, and 2 µg/mL venom concentration).

The heart weight of embryos was significantly increased compared to the control after the administration of the highest concentration of *C. adamanteus* venom (2000 µg/mL; *p* < 0.01) and after the administration of *C. atrox* venom at concentrations of 2 and 200 µg/mL (*p* < 0.01 for both). The other groups tested showed no significant change in embryonic heart weights compared to the control.

Lower liver weight was recorded only at the 20 µg/mL concentration of *C. adamanteus* venom compared to the control (*p* < 0.05). Higher concentrations of this venom did not influence the liver weights markedly. On the other hand, no tested concentration of *C. atrox* venom influenced embryonic liver weights significantly.

After the administration of *C. adamanteus* venom at a concentration of 200 µg/mL, one case of morphological alteration was observed, specifically the occurrence of Siamese twins, with one head having only one eye.

### 3.2. Vasoactivity

**Figure 1:**
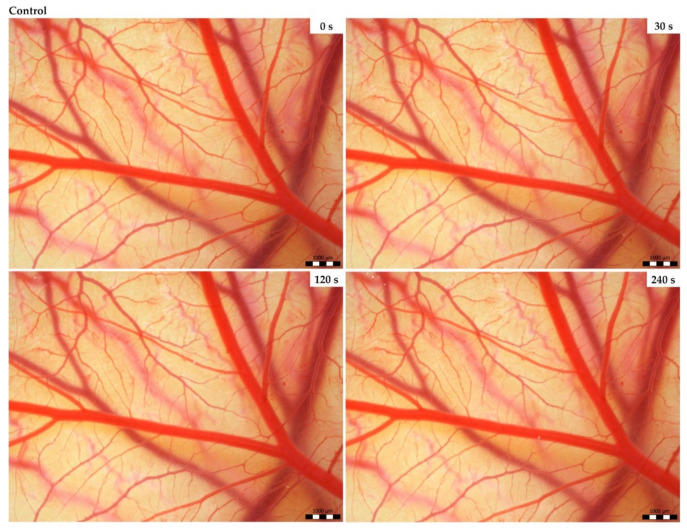
The selected results of the HET-CAM test for the control group in a monitored time frame. Note: Scale bar: 1 mm.

**Figure 2:**
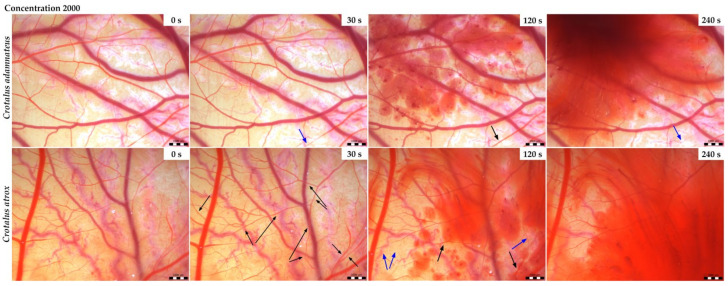
The selected results of the HET-CAM test for *Crotalus adamanteus* and *Crotalus atrox* venoms at 2000 µg/mL concentration in a monitored time frame. Note: Black arrow, vasodilation; blue arrow, vasoconstriction. Scale bar: 1 mm.

**Figure 3:**
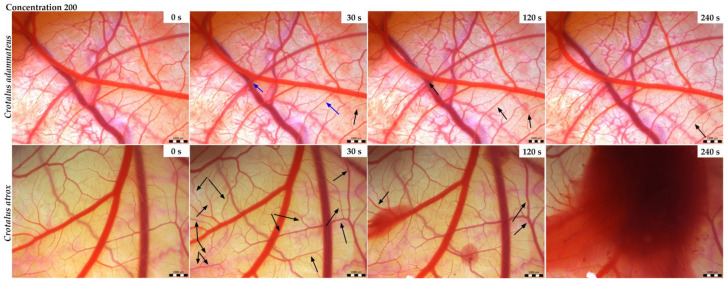
The selected results of the HET-CAM test for *Crotalus adamanteus* and *Crotalus atrox* venoms at 200 µg/mL concentration in a monitored time frame. Note: Black arrow, vasodilation; blue arrow, vasoconstriction. Scale bar: 1 mm.

In the control group (Figure 1) in which the sterile saline was applied, no toxic effects were observed. The effect of *Crotalus adamanteus* and *Crotalus atrox* venoms on the chorioallantoic membrane (CAM) had the same pattern with small temporal variations. For both venoms, hemorrhages of different intensities were observed depending on the venom type. Bleeding was preceded by either vasodilation or vasoconstriction depending on the concentration and type of the venom used. At higher concentrations (2000 µg/mL), occasional vasoconstriction was first observed 30 s after the application of *C. adamanteus* venom (Figure 2), followed by the dilation of vessels and capillaries associated with bleeding in the second minute. Interestingly, severe bleeding using this venom was noted after 240 s, with weak vasoconstriction occurring simultaneously at another site on the CAM. After the administration of *C. atrox* venom at a concentration of 2000 µg/mL (Figure 2), vasodilatation was observed at 30 and 120 s on the CAM, with occasional vasoconstriction and hemorrhage formation also noted after 120 s. Massive bleeding occurred 240 s after the administration of this venom. The hemotoxic effect on the membrane was considerably milder when venoms with lower concentrations (200 µg/mL) were used (Figure 3). Occasional vasoconstriction was observed on a portion of the CAM 30 s after the application of *C. adamanteus* venom, along with vasodilation, which continued throughout the measurement period. Severe bleeding was observed only with *C. atrox* venom after 240 s, which was preceded by the dilatation of vessels and capillaries, whereas only minimal bleeding was observed after the administration of *C. adamanteus* venom at a concentration of 200 µg/mL (Figure 3). After the application of both types of venom, no hyperemia or blood clotting was observed. All of the above changes on the CAM were observed within 5 min.

Luepke’s scoring system was used to calculate the irritant potential of the two venoms tested (Table 3) [30]. As hyperemia and blood clotting were not apparent, the average cumulative score resulted from the score of hemorrhages for each concentration of each venom type. Based on the cumulative scores and without regard to the concentration of venoms, the irritant potential of both venom types was classified as moderate at concentrations of 2000 µg/mL and as slight at 200 µg/mL.

Although both venoms with the same concentration of 2000 µg/mL started to act on the CAM surface at approximately the same time, the intensity of bleeding varied depending on the type of venom. The *C. atrox* venom had a more pronounced onset of bleeding within 120 s at a given concentration than *C. adamanteus* venom with the same concentration at the same time.

Even more pronounced differences in bleeding related to its onset and intensity were observed after the application of venoms with a concentration of 200 µg/mL. More pronounced bleeding with a faster onset of action at this concentration was observed on the vessel net after the application of *C. atrox* venom compared to *C. adamanteus* venom with the same concentration.

### 3.3. Acetylcholinesterase (AChE) Activity

**Figure 4:**
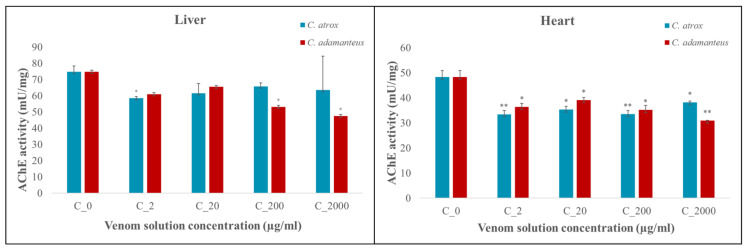
Acetylcholinesterase (AChE) activity in the embryonic liver and heart tissues after the application of *C. atrox* and *C. adamanteus* venoms. Note: Values represent the average ± SE (*n* = number of live embryos stated in Table 1). Statistically significant changes from controls are marked with an asterisk (* = *p* < 0.05; ** = *p* < 0.01).

After the administration of *C. adamanteus* and *C. atrox* venoms, AChE activity was observed in the embryonic liver and heart (Figure 4) tissues, and organ toxicity was subsequently determined. At all concentrations, an exclusively inhibitory effect of both venoms on AChE activity was observed.

After the application of *C. adamanteus* venom, there was a significant decrease in AChE activity in embryonic liver tissue at concentrations of 2000 µg/mL and 200 µg/mL (*p* < 0.05 for both), while in embryonic heart tissue, AChE was significantly inhibited at all concentrations (2000 µg/mL, *p* < 0.01 and 200, 20, and 2 µg/mL, *p* < 0.05).

The administration of 2 µg/mL *C. atrox* venom solution had an inhibitory (*p* < 0.05) effect on AChE activity in embryonic liver tissue. In the heart tissues, AChE activity was significantly inhibited at all concentrations of the venom solution (*p* < 0.05 for 2000 and 20 µg/mL and *p* < 0.01 for 200 and 2 µg/mL).

## 4. Discussion

Chicken embryos have long been considered the preferred experimental model due to their similarity in morphology, biochemistry, and genetics to humans and other mammals, making them ideal for drug screening and teratogen testing. Research on teratogens and their impact on fetal development, as well as human health risk assessments, is a key aspect of preclinical studies. The chick embryo model has proven to be useful in understanding embryonic development in humans, and its similarity to the human embryo is important for assessing the effects of different substances and their potential teratogenic effects [25]. The chicken embryo model is a readily available and cost-effective alternative animal model, finding its application in testing a variety of substances and materials. Its development takes place outside the mother’s body, allowing the effect of the test substance or biometrics to be observed directly on the embryo without any interference or detrimental effect on the mother’s metabolism [31]. Using chicken embryo models is also advantageous due to their availability and biological relevance to vertebrate development, allowing for an effective investigation of developmental toxicity. Considerable effort was devoted to the characterization and standardization of the CHEST (chick embryotoxicity screening test) protocol [32,33,34], which resulted in applying the test substance in small volumes of liquid in direct contact with the embryo. This approach makes it possible to monitor growth retardation, the occurrence of morphological changes, and mortality.

Using CHEST in the current study, high concentrations of the tested *C. adamanteus* venom resulted in a higher incidence of mortality in the chicken embryo than that of the *C. atrox* venom. The LD_50_ values for *C. adamanteus* venom (3.96 μg/egg) were almost three times lower than those determined for the *C. atrox* venom (11.73 μg/egg) indicating that the lower LD_50_ values reflect the higher level of the toxicity of the test venom and therefore a higher level of embryotoxicity. Years ago, a few studies dealt with the effect of *C. adamanteus* venom on animals’ mortality [35]. No studies, to our knowledge, dealt with such effects of *C. atrox* venom. Many recent publications have stated that venom toxicity varies between the species of the Viperidae family, which, among others, includes the Crotalus and Bitis genus. Petrilla et al. (2021) observed higher mortality and a lower lethal dose value (15.48 μg/egg) on chicken embryos after the application of *Bitis arietans* venom compared to *Bitis parviocula* venom (LD_50_ = 53.53 μg/egg) [28]. In the case of the Crotalus genus, similar to the current study, a concentration-dependent venom lethal effect of adult *Crotalus aquilus* (father), *Crotalus polystitans* (mother), and their juvenile hybrids (male/female) was observed with testing on mice [36]. In addition, *Crotalus durissus terrificus* venom at a dose of 200 µg/kg was shown to induce embryotoxicity as the fetuses showed abnormalities of the epiphyses and ribs [37]. Snake venom is a very complex mixture of different compounds, and its lethality is mainly due to the combination or synergy of enzymes and toxins [38]. High values in snake venom serine proteinase (SVSP) and phospholipase A2 (PLA2) and high variability of low molecular weight peptides (probably crotamine-like peptides) were found to relate to their toxicity [36]. In a recent study [39], the venom of *Crotalus pricei pricei* was slightly more toxic to geckos than that of *Crotalus tancitarensis*, and LD_50_ did not exceed 1 µg/g body weight. In other work, for comparison, venom LD_50_ of the same species, *C. p. pricei* (Chiricahuas), was 1.25 μg/g when tested on mice [40]. The treatment of *Crotalus basiliscus* envenomation mandates several different antivenoms to neutralize specific toxins in different venom compositions of only one species [9]. This shows that lethality (LD_50_) within species varies with the composition of venom, which can lead to health complications with varying symptomatology or ineffective antivenom treatment.

The snake families Viperidae and Elapidae, which share several of the same proteins and peptides, have attracted the most research attention. However, their biological activity may differ as the chemical composition of venom varies by family, genus/species, the geographical location of the snake, prey type, and theage or size of the individual [41]. Snakes belonging to the subfamily Crotalinae inhabit diverse habitats in America, ranging from deserts, scrublands, and savanna or grassland biomes to coastal areas [6,9]. Their habitats also vary in elevation, food type, and geographic range, allowing them to exhibit considerable variation in venom composition and actions [9].

Envenomation by Crotalus species is characterized by neurological, myotoxic, and nephrotoxic syndromes that manifest in varying intensity depending on the amount of venom, the weight of the victim, and the size of the snake [42]. The current study revealed markedly heavier chicken embryos and their hearts after the application of *C. atrox* venoms than after the application of *C. adamanteus* venom. In addition, after the application of *C. adamanteus* venom at a concentration of 200 μg/mL, morphological changes were observed; specifically, in one case of Siamese twins with only one eye dominant on one head. Many authors attribute these effects of bite injuries from *C. atrox* on structural disruptions of victim’s organs to the proteolytic activity of snake venom metalloproteinases (SVMPs) [43] and disintegrins [44,45]. Chorioallantoic membrane (CAM) has proven to be a suitable agent in experimental studies because the tissue responses of CAM are similar to those found in mammals, and its response in toxicology studies resembles those in other animal models [26]. Chick embryo CAM provides a valuable model for imaging changes in blood vessels and vascular damage. Both venoms tested (*C. adamanteus* and *C. atrox*) demonstrated the ability to induce changes in the vascular network in chick CAM. The hemotoxicity of both tested venoms has not yet been confirmed and documented in any work. However, a recently published study [9] compared the venom composition of the genus Crotalus representatives, including *C. adamanteus* and *C. atrox*, and documented 63 protein families and subfamilies, including major hemotoxic enzymes, such as snake venom serine protease (SVSP), snake venom hyaluronidase (SVH), snake venom metalloprotease (SVMP), and phospholipase A2 (PLA2). In the current study, selected *Crotalus venoms* (*C. adamanteus* and *C. atrox*) were applied on the CAM surface to ascertain their potential hemorrhagic effect. Massive hemorrhage was preceded by vasodilation or occasional vasoconstriction in both types of venoms in a dose-dependent manner, with varying intensities and a slight-to-moderate irritant potential over 5 min. Generally, the Viperidae snake venoms have mostly vasodilating effects caused by PLA2 fractions depending on the amount and activity of the enzyme [9]; thus, we are assuming that it could have the same effect in our case. To date, there are no data describing the vasoconstrictive effect of venoms; therefore, this will be the subject of further research. The application of *C. adamanteus* exhibited greater toxicity, and *C. atrox* caused more pronounced bleeding with a faster onset of action, which may relate to more hemotoxic components in *C. atrox* venom. Knight et al. [26] observed hemostatic changes induced by the application of *Crotalus viridis* venom to the CAM surface, noting heavy bleeding and clots as early as 30 s after the venom was administered at the E-2 concentration (the venom was diluted with sterile distilled water to give equal concentrations based on molecular weights). In addition, the authors tested the venoms of two other viper snakes (*Agkistrodon contortrix* and *Bitis arietans*) along with *Crotalus viridis*, revealing bleeding and clotting too. As in our study, no hyperemia was observed, and the irritant potential of all three venoms was very similar. Although with a more dilute concentration of E-3 venom the effects did not become apparent until 120 s, both concentrations were characterized by a strong irritant potential. Viper family venoms are characterized by the action of zinc-containing metalloproteinases, which cause the degradation and breakdown of proteins in the capillary endothelium. These effects lead to both local and systemic hemorrhages, which can result in death due to cardiovascular shock [46]. Moreover, the Crotalus snake venom intoxication alters the blood clotting system. These effects are probably due to the presence of enzymes, such as the aforementioned serine proteases and metalloproteinases, which have a primary effect on the hemostatic system [14]. The hemorrhagic effects of *C. atrox* venom has been tested on human embryonic kidney cells (HEK 293T), in which the venom induced a strong oxidative response in those cells and exhibited significant cytotoxicity [43]. Cell clumping and fragmentation were observed after the application of higher concentrations of *C. atrox* venom (100 and 500 μg/mL). Baldo et al. [47] observed that toxic reactions caused by hemorrhagic snake venom include local tissue destruction. In addition, cardiotoxic effects have been demonstrated in horses that have been bitten by snakes of the genus Crotalus as well as in experimental animals that have been administered the venom of *Crotalus durissus cumanensis* [48]. In this study, mild effects in the form of tumefaction on cardiac fibers and vacuolization were observed after the application of *Crotalus vegrandis* venom, which can affect the cardiac action potential conduction and cause electrocardiographic disturbances. Envenomation by Crotalidae snakes, including rattlesnakes, can be life-threatening to both the fetus and mother in almost half of pregnant women. The bite causes hemorrhagic diathesis due to the procoagulant activity of these venoms [37]. In this study, the subcutaneous administration of two different doses of *Crotalus durissus terrificus* venom (75 µg/kg; 200 µg/kg) on day 3 of mouse gestation resulted in mild maternal and fetal toxicity only at the higher dose of the venom. A new rabbit (New Zealand white) model was used by Nielsen, Vance G. [49] to characterize the toxicodynamic profile of coagulopathy caused by *C. atrox* venom at a dose of 4 mg/kg administered subcutaneously. Significant deterioration in clot formation rate and clot strength was observed in animals within 3 h [49].

Crotalus venom (*C. durissus terrificus*) was also found to cause acute hepatotoxicity [50] with increased levels of liver enzymes (ALT, AST, ALP, and GGT) that may be related to tissue damage [14].

A recent study analyzing the venom composition of the genus Crotalus indicates the presence or absence of active acetylcholinesterase (AChE) in the venom, with it being absent in selected species such as *C. adamanteus* and *C. atrox* [9]. Therefore, it can be assumed that the measurement of AChE activity was not influenced by AChE in venom but was the result of the interaction of venom components with the analyzed AChE levels in embryonic tissues related to tissue damage [14]. The present work newly reports an inhibitory effect of selected *Crotalus venoms* (*C. adamanteus* and *C. atrox*) on AChE activity observed in the embryonic heart and liver tissues in a dose-dependent manner. We found that AChE activity varied in chick embryos at different venom concentrations. An inhibitory effect on AChE activity was observed in the same embryonic tissues (liver, heart) affected by venom from *Bitis parviocula* and *Bitis arietans*. However, venom from *Bitis arietans* showed alternating inhibitory or stimulatory effects on AChE activity depending on the venom concentration [28]. Why the AChE activity at different venom concentrations varied is not known and indicates the need for further studies to analyze the different components of Crotalus snake venom and to determine which substances can be attributed to having an inhibitory or stimulatory effect on AChE activity.

## 5. Conclusions

The study evaluated the embryotoxic effects of snake venoms from *Crotalus adamanteus* and *Crotalus atrox* species on the development of chicken embryos using the chick embryotoxicity screening test (CHEST) and the chorioallantoic membrane assay (HET-CAM). The results suggest that snake venoms may significantly affect embryonic development and the health of chicken embryos. *C. adamanteus* venom application revealed higher embryotoxicity and morphological abnormalities, such as Siamese twins, and exhibited greater toxicity. The administration of *C. atrox* venom caused more pronounced bleeding with a faster onset of action, which may relate to more hemotoxic components in *C. atrox* venom. Furthermore, the application of both venoms induced changes in the whole embryo, heart, and liver weights as well as massive hemorrhages in a dose-dependent manner with various intensities and a slight-to-moderate irritant potential. Specific concentrations of the venoms inhibited acetylcholinesterase activity in the liver and heart tissues of embryos. These results highlight the need for further studies and a better understanding of the toxicological effects of Crotalus snake venoms on different tissues and organisms.

## Figures and Tables

**Table 1:** The effects of *Crotalus adamanteus* and *Crotalus atrox* venoms on embryo development obtained with the CHEST.

Snake Venom	Conc. (µg/mL)	*n*	Live Embryos	Dead Embryos	Mortality (%)
**None (Control)**	**0**	13	13	0	0
***Crotalus adamanteus***	**2**	13	12	1	7.69
	**20**	13	11	2	15.38
	**200**	13	10	3	23.07
	**2000**	13	10	3	23.07
***Crotalus atrox***	**2**	13	13	0	0
	**20**	13	11	2	15.38
	**200**	13	12	1	7.69
	**2000**	13	12	1	7.69

Note: *n*, number of embryos used.

**Table 2:** Weight changes were observed on ED9 of the CHEST after applying individual concentrations of *Crotalus adamanteus* and *Crotalus atrox* venom.

Snake Venom	Conc. (µg/mL)	Embryo Weight (g)	Heart Weight (g)	Liver Weight (g)
**None (control)**	**0**	0.886 ± 0.014	0.007 ± 0.000	0.010 ± 0.001
***Crotalus adamanteus***	**2**	0.898 ± 0.023	0.007 ± 0.000	0.010 ± 0.001
	**20**	0.897 ± 0.028	0.007 ± 0.001	0.009 ± 0.001 *
	**200**	0.887 ± 0.053	0.007 ± 0.001	0.010 ± 0.001
	**2000**	0.819 ± 0.032 *	0.009 ± 0.001 **	0.009 ± 0.001
***Crotalus atrox***	**2**	1.047 ± 0.026 ***	0.008 ± 0.001 **	0.012 ± 0.001
	**20**	1.078 ± 0.050 ***	0.007 ± 0.001	0.012 ± 0.001
	**200**	1.093 ± 0.028 ***	0.008 ± 0.001 **	0.013 ± 0.001
	**2000**	1.002 ± 0.033 **	0.009 ± 0.001	0.009 ± 0.001

Note: Values represent the average ± SE (*n* = number of live embryos stated in Table 1). Statistically significant changes from the control are marked with an asterisk (* = *p* < 0.05; ** = *p* < 0.01; *** = *p* < 0.001).

**Table 3:** The classification of *Crotalus adamanteus* and *Crotalus atrox* venoms according to the evaluation of the irritant potential.

Snake Venom	Conc. (µg/mL)	Hyperemia	Hemorrhage	Blood Clotting	Total Average (Cumulative Score)	Irritant Potential
**None (Control)**	**0**	0	0	0	0	0
***Crotalus adamanteus***	**200**	0	3.5	0	3.5	Slight
	**2000**	0	5.5	0	5.5	Moderate
***Crotalus atrox***	**200**	0	4.0	0	4.0	Slight
	**2000**	0	5.0	0	5.0	Moderate

Note: Values represent the average ± SE (n = 4 for each venom and each concentration).

## Data Availability

Additional data are available on request from the corresponding author.

## Supplementary Materials

The following supporting information can be downloaded at https://www.mdpi.com/article/10.3390/ani14111634/s1. Figure S1: Images used for evaluation of the HET-CAM test for *Crotalus adamanteus* venom at 2000 µg/mL concentrations in a monitored time frame; n = 4; Figure S2: Images used for evaluation of the HET-CAM test for *Crotalus adamanteus* venom at 200 µg/mL concentrations in a monitored time frame; n = 4; Figure S3: Images used for evaluation of the HET-CAM test for *Crotalus atrox* venom at 2000 µg/mL concentrations in a monitored time frame; n = 4; Figure S4: Images used for evaluation of the HET-CAM test for *Crotalus atrox* venom at 200 µg/mL concentrations in a monitored time frame; n = 4.
