# Supplementary material for: A Comparative Analysis of the Cytotoxic and Vascular Activity Effects of Western Diamondback Rattlesnake (Crotalus atrox) and Eastern Diamondback Rattlesnake (Crotalus adamanteus) Venoms Using a Chick Embryo Model
Source: Animals (Basel). 2024 May 30;14(11):1634. doi: 10.3390/ani14111634 (PMC11171291; doi:10.3390/ani14111634)
Supplement: Supplementary file 1 [file animals-14-01634-s001.zip › animals-3020162-supplementary.pdf]

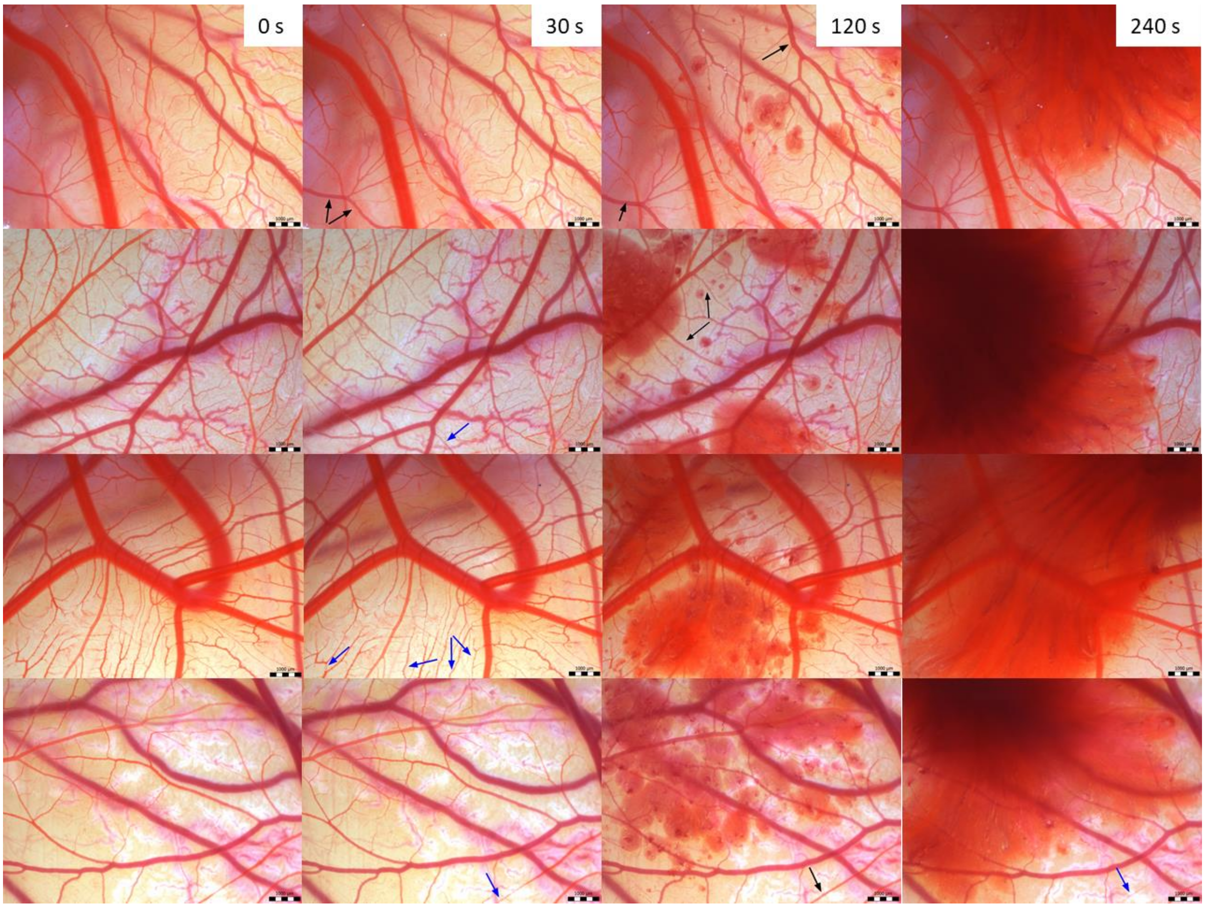

Figure S1: Images used for evaluation of the HET-CAM test for Crotalus adamanteus venom at 2000 µg/ml concentrations in a monitored time frame; n=4

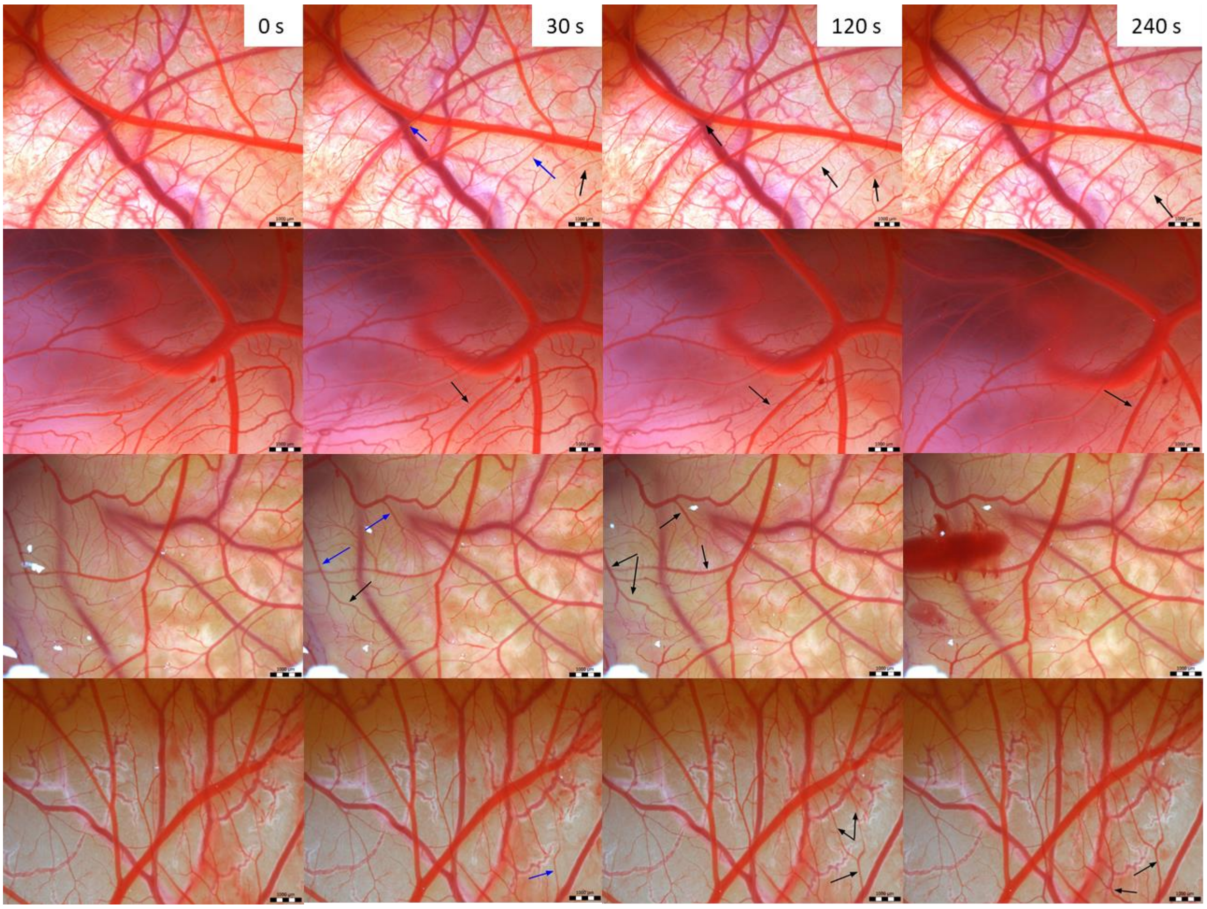

Figure S2: Images used for evaluation of the HET-CAM test for Crotalus adamanteus venom at 200 μg/ml concentrations in a monitored time frame; n=4

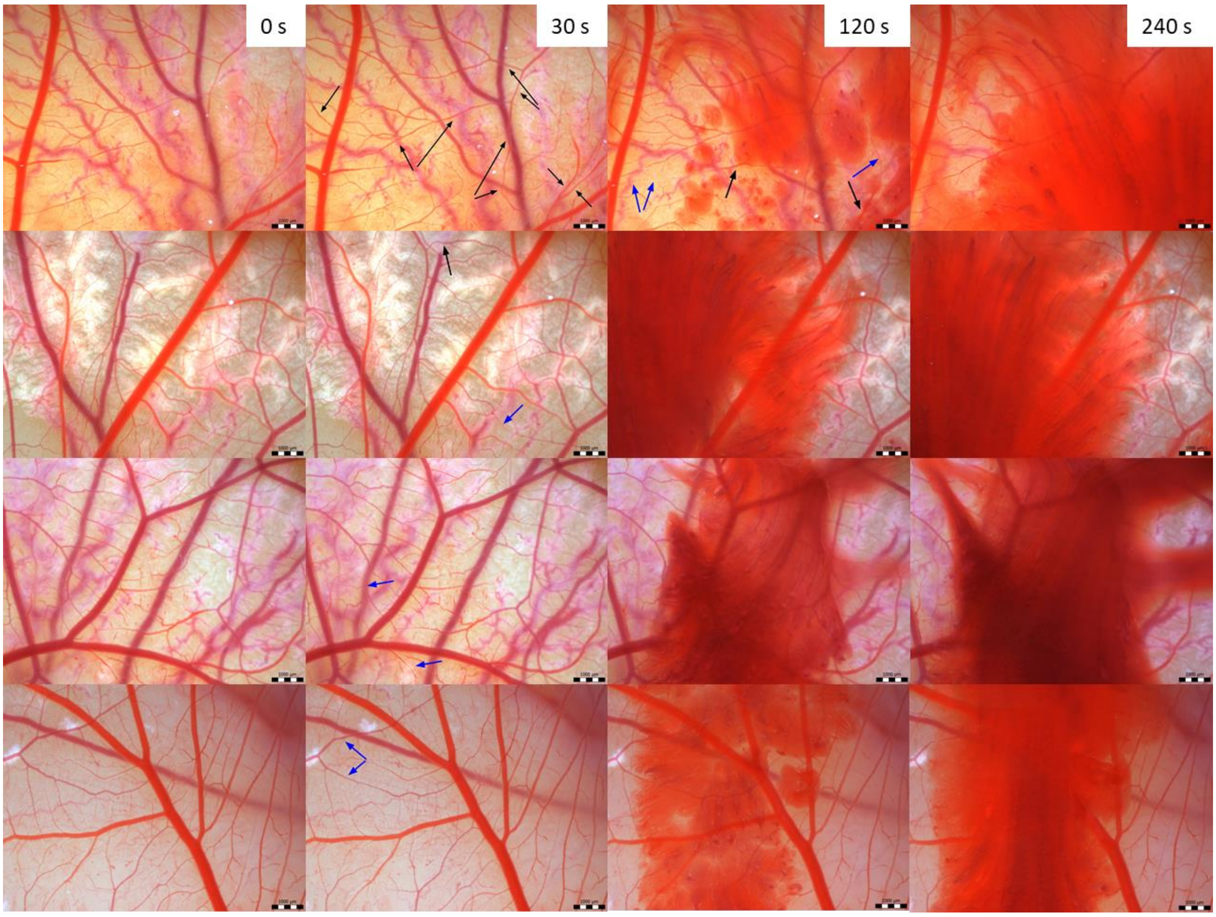

Figure S3: Images used for evaluation of the HET-CAM test for Crotalus atrox venom at 2000 μg/ml concentrations in a monitored time frame; n=4

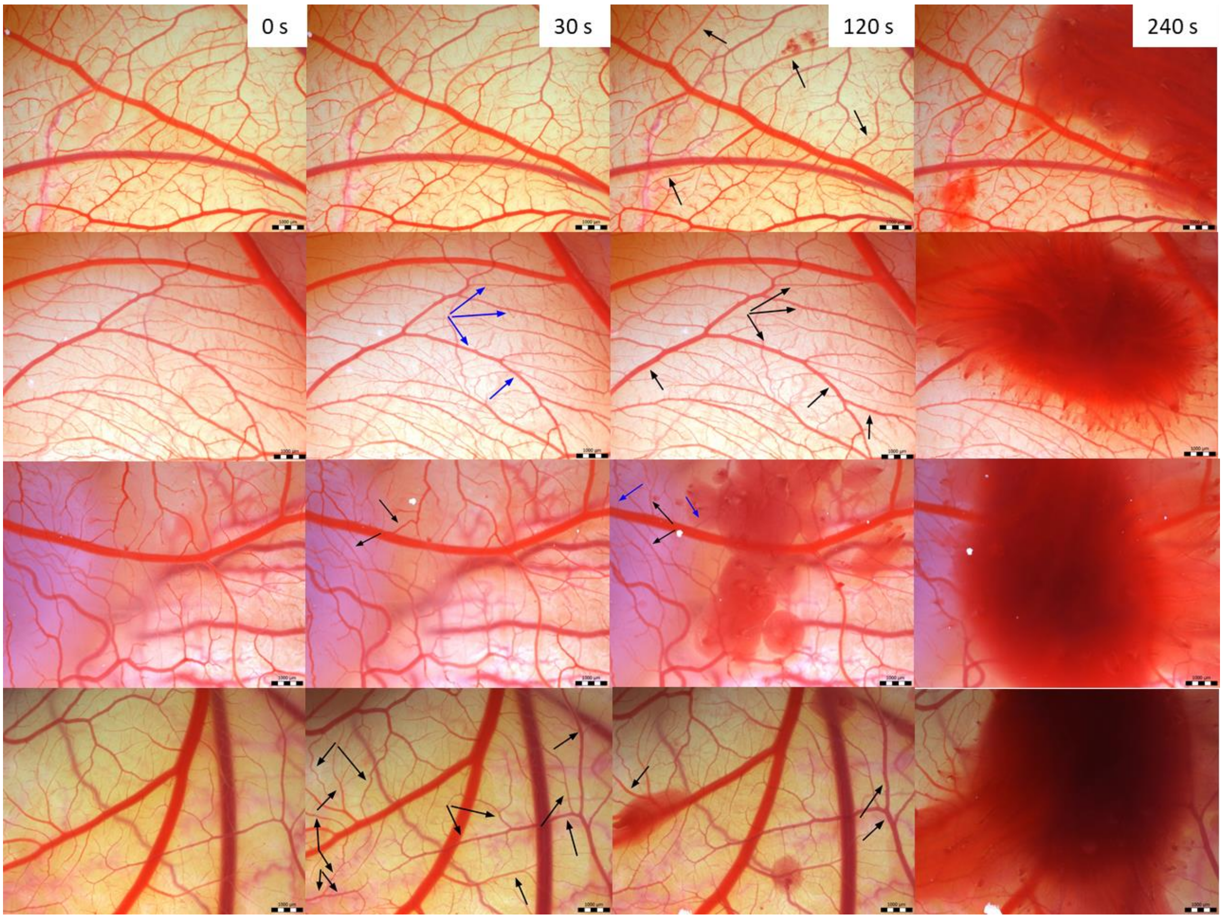

Figure S4: Images used for evaluation of the HET-CAM test for Crotalus atrox venom at 200 µg/ml concentrations in a monitored time frame; n=4
